# Combined Hamartoma of the Retina and Retinal Pigment Epithelium: Multimodal Imaging Characterization of an Epipapillary Case Presenting With Severe Visual Loss

**DOI:** 10.7759/cureus.109904

**Published:** 2026-05-29

**Authors:** Daniele Brocca, Alessandro Franceschi, Alberto Bonato, Nicola Zemella

**Affiliations:** 1 Ophthalmology, De Gironcoli Hospital, Conegliano, ITA; 2 Ophthalmology, University of Padua, Padova, ITA

**Keywords:** combined hamartoma retina rpe, epipapillary hamartoma, multimodal retinal imaging, optical coherence tomography, vitreoretinal traction

## Abstract

Combined hamartoma of the retina and retinal pigment epithelium (CHRRPE) is a rare benign lesion that may present with marked vitreoretinal distortion and simulate other tractional or exudative retinal disorders. A 53-year-old woman was referred to the ophthalmic emergency department for suspected retinal detachment in the left eye and presented with hand-motion visual acuity. Multimodal imaging, including infrared reflectance, blue reflectance, blue-light autofluorescence, multicolor imaging, fluorescein angiography (FA), and spectral-domain optical coherence tomography (SD-OCT), demonstrated a large epi-papillary fibroglial lesion with severe posterior pole distortion. Infrared and multicolor imaging highlighted radially dragged retinal vessels, fibrogliotic extensions spreading temporally from the lesion, and marked corrugation of the posterior pole. Blue-light autofluorescence showed a predominantly hypoautofluorescent mass with multiple focal hyperautofluorescent areas at the temporal margin, corresponding to areas of maximal traction. FA revealed early masking hypofluorescence followed by progressive staining of intrinsic fibrovascular tissue and abnormal vascular loops at the disc margin, without peripheral anomalies. SD-OCT was crucial in clarifying the structural changes, disclosing a massive dome-shaped epipapillary elevation, full-thickness retinal disorganization, broad splitting between inner and outer retinal layers, multiple schitic cavities, and a temporally located posterior pole sector of abnormally hyperreflective, disorganized inner retinal layers compatible with chronic traction‑related ischemia. This case emphasizes the value of multimodal imaging in detecting remarkable retinal alterations in a case of advanced epipapillary CHRRPE. Accurate cross-correlation among imaging modalities is essential for diagnosis, prognosis, and surgical decision-making.

## Introduction

Combined hamartoma of the retina and retinal pigment epithelium (CHRRPE) is a rare, benign, congenital proliferative lesion first described by Gass in 1973 as an unusual hamartoma of the pigment epithelium and retina simulating choroidal melanoma and retinoblastoma [1]. It is characterized by a disorganized, non-progressive proliferation of multiple retinal cell types, including retinal glial cells, vascular elements, and retinal pigment epithelial (RPE) cells, forming an elevated, ill-defined grayish lesion that distorts the underlying retinal architecture. Unlike isolated congenital hypertrophy of the retinal pigment epithelium (CHRPE), which is typically flat and clinically inert, CHRRPE may present with marked retinal distortion, vascular tortuosity, epiretinal membrane formation, and severe visual impairment. [2,3].

The lesion preferentially involves the posterior pole, with the optic disc and peripapillary region representing the most frequent anatomical location (approximately 50-60% of cases), followed by the macula and mid-peripheral retina [4]. In large series, the annual incidence is estimated at less than one per million, and there is no definitive sex predilection [5]. Although typically sporadic, an association has been reported with neurofibromatosis type 1 (NF1), neurofibromatosis type 2 (NF2), and tuberous sclerosis complex [6,7].

The clinical diagnosis may be challenging, particularly in advanced or atypical presentations, given the overlapping features with other epipapillary lesions such as astrocytic hamartomas, choroidal melanoma, retinoblastoma, juxtapapillary choroidal neovascularization, epiretinal membranes, and papilledema [3,8]. In this context, multimodal imaging has fundamentally improved diagnostic tools and lesion characterization.

Epipapillary CHRRPE represents a diagnostic and prognostic challenge, particularly when presenting with severe visual loss in adulthood, a setting in which the full extent of structural damage may be underappreciated without systematic multimodal imaging. We report a case of epipapillary CHRRPE to document the complete multimodal imaging correlates of an exceptionally advanced lesion, characterizing novel structural features not previously described in their entirety in this condition, and highlighting the diagnostic and prognostic value of cross-modal image correlation in this clinical setting, encompassing infrared reflectance (IR), blue-light autofluorescence (BAF), multicolor fundus imaging, fluorescein angiography (FA), and spectral-domain OCT (SD-OCT).

## Case presentation

A 53-year-old Caucasian female was referred to the ophthalmic emergency department, Ospedale di Conegliano “De Gironcoli”, Treviso, Italy, in March 2026 for suspected retinal detachment in the left eye. The patient was unable to specify the duration of visual loss and denied any prior systemic diagnoses, trauma, or ophthalmological interventions. Best-corrected visual acuity (BCVA) was 10/10 in the right eye and hand motion in the affected eye (LE). Anterior segment examination by slit-lamp biomicroscopy was unremarkable bilaterally. Intraocular pressure was within normal limits. Fundus examination of the LE revealed a prominent fibroglial lesion, firmly adherent to the optic disc, exerting traction on the surrounding tissue. Peripheral retina and fundus examination of the fellow eye were unremarkable. A working diagnosis of combined hamartoma of the retina and retinal pigment epithelium (CHRRPE) was suspected, and therefore, the patient was scheduled for a complete multimodal imaging workup.

**Figure 1:**
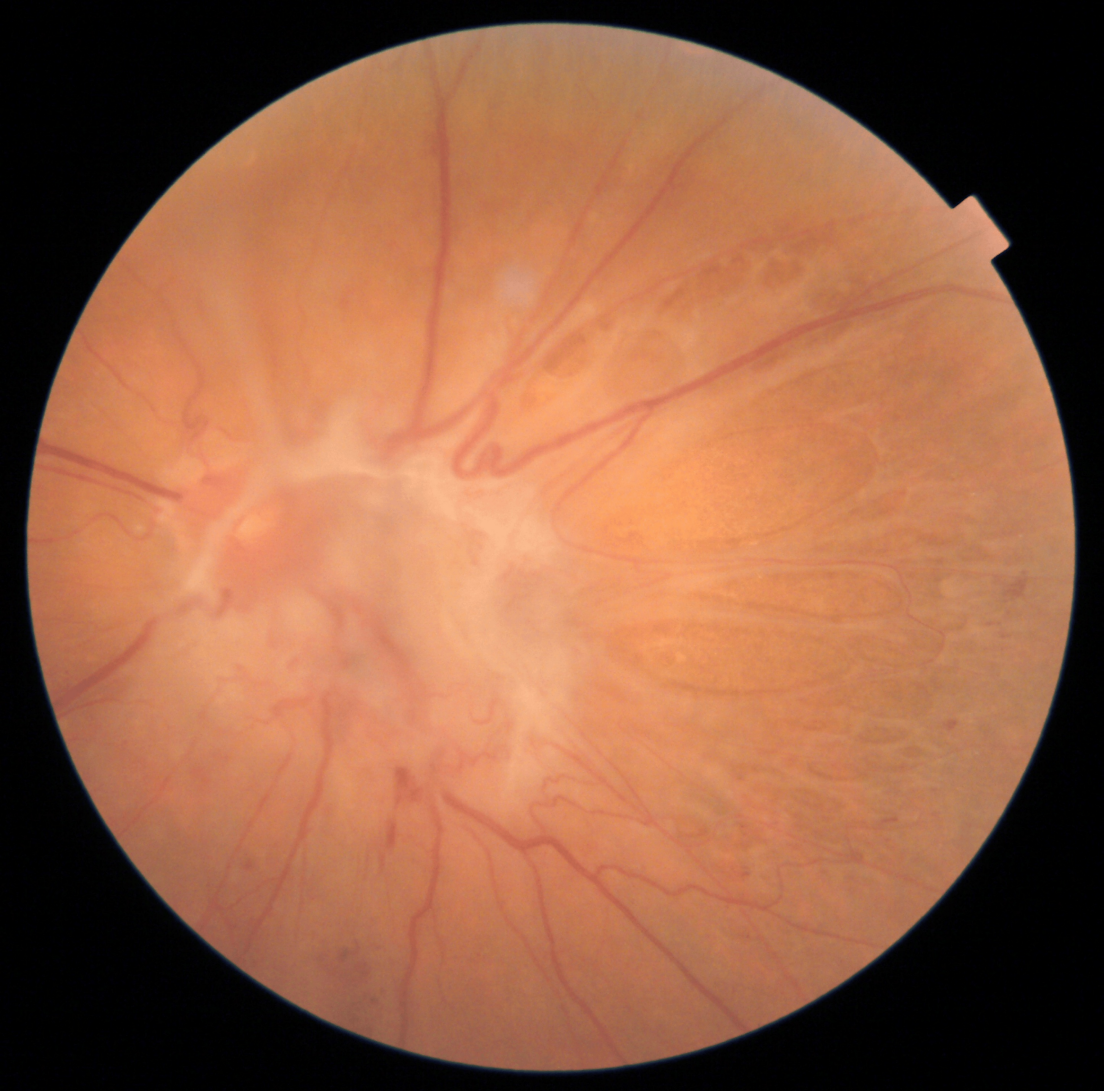
Color fundus photograph of the left eye. Color fundus photograph of the left eye showing a large epipapillary fibrovascular complex extending along the papillomacular bundle, with distortion of the superficial retinal vasculature, macular inner‑retinal loss from advanced retinoschisis, and scattered inferior mid‑peripheral hemorrhages.

A color fundus photograph of the LE acquired with a confocal scanning laser ophthalmoscope, Topcon DRI Triton (Topcon Corp., Tokyo, Japan), showed an extensive fibrovascular complex centered on the optic disc and extending along the papillo-macular bundle (Figure 1). The lesion appeared as a whitish, elevated, ill‑defined plaque in which epipapillary fibrous tissue was tightly interwoven with the superficial neurosensory retina, encasing and distorting the overlying retinal vasculature, including several major vessels. Within the macular region, multiple apparent “holes” corresponded not to full‑thickness macular defects, but to areas where the inner retinal layers were absent, creating sharply demarcated zones of exposed outer retina. In addition, scattered intraretinal hemorrhages were observed in the inferior mid‑periphery, further reflecting the severity of the tractional and microvascular alterations associated with the hamartomatous complex.

Other imaging was performed using a confocal scanning laser ophthalmoscope (Heidelberg Engineering Spectralis, Heidelberg, Germany).

**Figure 2:**
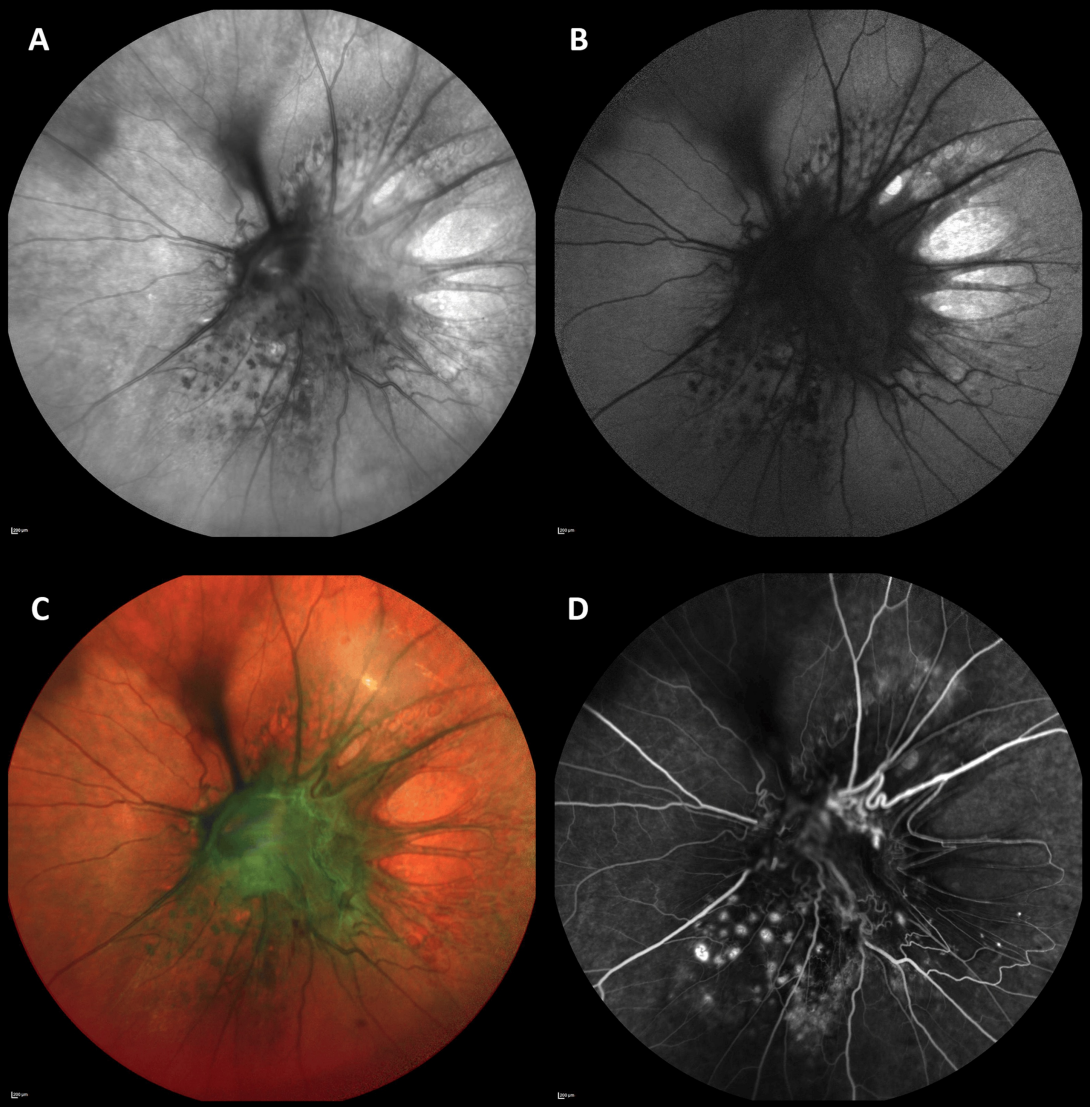
Multimodal imaging of the left eye. (A) Infrared reflectance shows a hyporeflective star‑shaped epipapillary mass extending along the papillomacular bundle, with additional hyporeflective spots in the mid‑periphery and temporal macular hyperreflectivity corresponding to inner‑retinal tissue loss and schisis. (B) Blue‑light autofluorescence demonstrates a predominantly hypoautofluorescent star‑shaped lesion with a homogeneous hyperautofluorescent temporal macular plaque corresponding to pseudoholes. (C) Multicolor imaging reveals a green‑dominant epipapillary complex completely obscuring the optic disc and papillomacular bundle, with inferior parapapillary pinpoint changes and temporal macular pseudoholes, as well as a funnel‑shaped epiretinal tractional prominence protruding into the anterior vitreous. (D) Fluorescein angiography showing a hypofluorescent epipapillary mass with intrinsic tortuous vascular loops, multiple hyperfluorescent foci at the inferior margin, preserved peripheral perfusion, and subtly reduced capillary detail with a sectoral perfusion mismatch at the temporal posterior pole.

Infrared reflectance and blue-light autofluorescence imaging delineated the full extent of the fibroglial lesion and its tractional consequences: the star-shaped hyporeflective epipapillary mass, the mid-peripheral hyporeflective spots, and the temporal hyperautofluorescent oval areas, corresponding to zones of inner retinal tissue loss and pseudohole formation from advanced retinoschisis, collectively reflected the severity of tangential traction exerted by the hamartomatous complex (Figures 2A, 2B). Multicolor imaging added interpretive value by highlighting the green-dominant discoloration of the lesion through the 518 nm channel, consistent with altered vitreoretinal interface reflectance from fibrous and glial tissue, and by rendering the funnel-shaped epiretinal tractional prominence and the schitic inner retinal areas in the temporal sector as regions of maximal tangential stress (Figure 2C). Fluorescein angiography demonstrated early masking hypofluorescence of the epipapillary mass, progressive staining of the intrinsic fibrovascular tissue with increasingly conspicuous tortuous vascular loops at the disc margin, and a notable sectoral perfusion mismatch at the temporal posterior pole, a finding with potential implications for chronic ischemic inner retinal remodeling in this region (Figure 2D).

High-speed SD OCT was performed covering multiple radial and horizontal sections through the optic disc and posterior pole, demonstrating findings of exceptional severity.

**Figure 3:**
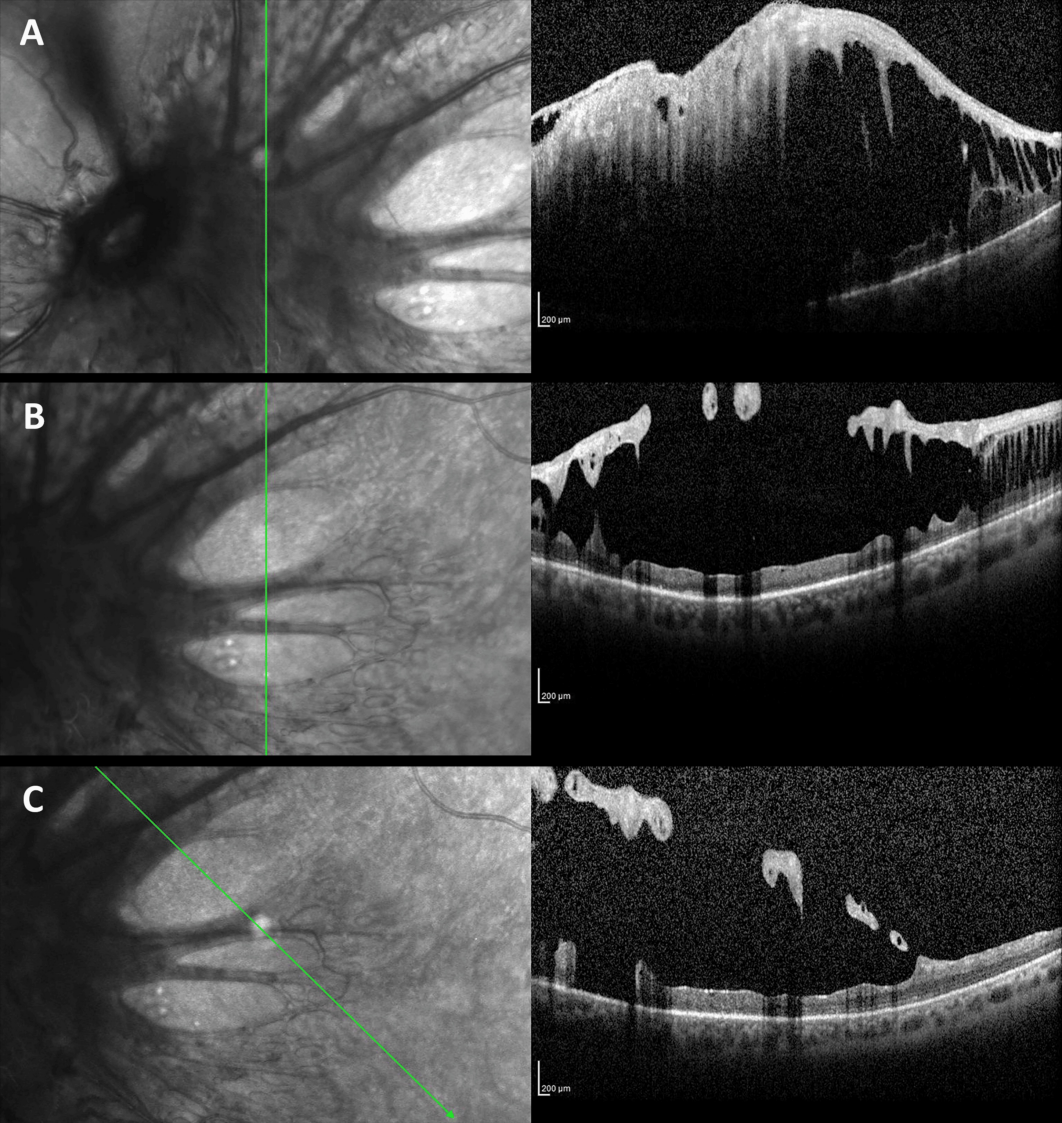
Spectral‑domain OCT of the left eye. (A) Vertical scan through the optic disc showing a large dome‑shaped epipapillary mass composed of a hyperreflective epiretinal band with intralesional hyporeflective spaces. (B) Vertical macular scan demonstrating complete splitting between inner and outer retinal layers, with extensive retinoschisis, focal absence of inner retinal tissue and imbrication of the inner retinal leaf with hyperreflective epiretinal material. (C) Oblique macular scan again illustrating inner–outer retinal splitting with areas of missing inner retina and an inner retinal macular hole in the superonasal macula.

The dominant feature was a massive dome-shaped epipapillary elevation, corresponding to the full-thickness fibroglial mass adherent to and centered on the optic disc head. The inner retinal surface was irregular and hyper-reflective, consistent with a thickened epiretinal membrane-like fibrovascular tissue. Full-thickness retinal architectural disorganization was evident throughout the lesion, with loss of the normal laminar retinal structure (Figure 3A). A vertical SD-OCT image passing through the macula showed a complete splitting between the inner retinal layers and the outer retinal layers (retinoschisis). Moreover, further linear scans passing through the macula showed a complete absence of inner retinal tissue (Figure 3B, 3C). In addition, an inner retinal macular hole can be observed at the level of the superonasal macula.

Based on the clinical examination and multimodal imaging findings, the differential diagnosis included epipapillary CHRRPE as the leading diagnosis, with optic disc melanocytoma, reactive epipapillary gliosis, and juxtapapillary choroidal neovascularization considered and excluded: melanocytoma was ruled out by the absence of a deeply pigmented, homogeneously hyporeflective disc mass and by the presence of full-thickness inner retinal involvement with vitreoretinal traction on SD-OCT; reactive gliosis and juxtapapillary choroidal neovascularization were excluded based on the characteristic fibrovascular staining pattern on FA and the absence of a subretinal neovascular complex. Retinal detachment was excluded by the absence of a rhegmatogenous or exudative component and the confirmed retinal adherence on clinical examination and imaging. Given the extent of structural damage and the severity of visual loss at presentation, a conservative approach was initially adopted, with the patient counselled regarding the guarded visual prognosis. Surgical planning, including pars plana vitrectomy with epiretinal fibrovascular tissue peeling, is currently under evaluation.

## Discussion

CHRRPE is a rare, congenital, non-hereditary lesion of uncertain histogenesis. Although first recognized by Gass in 1973 [1], the largest clinical series to date was published by Shields et al. in 2020, which reviewed 77 consecutive patients and provided the most comprehensive characterization of clinical and demographic features [4]. In this series, the mean age at diagnosis was 15.4 years (range 0-84), though cases presenting in adulthood - as in the present report - are well documented and tend to be larger and more symptomatic at the time of detection, likely due to delayed diagnosis [4,9]. The age of our patient at presentation - 53 years - is notably higher than the mean age at diagnosis and is consistent with the subset of adult-onset cases characterized by delayed diagnosis and more advanced structural damage at the time of detection. A plausible explanation for the protracted asymptomatic course in our patient lies in the laterality of the affected eye: the left eye, presumably the non-dominant eye, may have undergone progressive visual deterioration over the years without triggering significant functional complaints in daily activities, particularly given the preserved visual acuity in the fellow eye. 

Building on the differential diagnosis outlined at clinical presentation, multimodal imaging allowed systematic and confident exclusion of the main epipapillary mimickers. Optic disc melanocytoma was excluded based on the absence of a deeply pigmented hyporeflective disc mass and the presence of full-thickness inner retinal involvement with vitreoretinal traction on SD-OCT, features incompatible with a purely pigmented choroidal lesion. Peripapillary choroidal tumors were ruled out by the absence of any homogeneous subretinal or choroidal mass component on both SD-OCT and FA. Papilledema was excluded by the lack of disc elevation attributable to raised intracranial pressure and by the characteristic fibrovascular staining pattern on FA. The green-dominant inner retinal signal on multicolor imaging, reflecting altered vitreoretinal interface properties rather than a pigmented or choroidal process, further supported CHRRPE as the only entity accounting for the complete multimodal picture [2,3].

Histopathologically, CHRRPE consists of a disorganized proliferation of retinal glial cells, RPE cells, blood vessels, retinal neurons, and fibrous tissue, arranged without normal architectural lamination [10]. The epipapillary and peripapillary variant, as seen in our patient, is characterized by a fibrovascular epiretinal membrane component that is particularly prone to causing progressive vitreoretinal traction and reduced visual acuity over time [4,5].

Visual outcomes in epipapillary CHRRPE are typically poor, particularly in cases presenting with macular traction, and BCVA at the level of hand motion, as documented in our patient, has been reported in large or long-standing lesions [5]. Nevertheless, Cohn et al. [11] showed architectural improvement after vitrectomy with plasmin of ERM-associated CHRRPE lesions, with significant visual improvement of four of six pediatric eyes. SD-OCT has fundamentally improved the understanding of CHRRPE, enabling in vivo characterization of its inner retinal architecture and vitreoretinal relationships.

The first systematic OCT description was provided by Shields et al. in 11 consecutive cases, identifying inner surface irregularity, full-thickness retinal thickening, loss of normal laminar architecture, and preretinal membrane as the most consistent features [12]. Subsequent work by Chawla et al. [13] in 16 macular CHRRPE eyes refined this understanding, demonstrating that the lesion is fundamentally a hamartoma of the inner retinal layers, with the outer plexiform layer (OPL) acting as the typical posterior boundary of tissue involvement. In that series, the OPL showed a characteristic "saw-tooth" configuration - sharp-peaked intraretinal undulations generated by the contracted fibro-vascular tissue - in 75% of cases, while a subset of lesions demonstrated the more pronounced "omega sign," in which the OPL convolutions are broader and deeper, forming a sinuous Ω-shaped profile on cross-section; this latter finding was further characterized as a distinct OCT marker of macular CHRRPE by Kumar et al. [14] In our case, the epipapillary location and the exceptional size of the lesion resulted in a structural severity that goes substantially beyond these macular phenotypes. Full-thickness retinal disorganization extended throughout the mass without any recognizable laminar boundary, and no intact OPL was identifiable in the scan planes crossing the lesion core, consistent with the more severe structural disruption associated with peripapillary versus macular location, as highlighted in the comparative imaging analysis by Gupta et al., who documented a higher frequency of full-thickness retinal involvement, RPE disruption, and outer retinal attenuation in peripapillary compared to macular CHRRPE [15]. The degree of vitreoretinal traction, with thickened posterior hyaloid adherence, vertical tractive bands, and consequent retinal schitic modifications, is likewise consistent with the severe end of the CHRRPE spectrum, and directly accounts for the BCVA of hand motion at presentation. Arrigo et al. [16], in their combined OCT and OCT-angiography analysis, similarly emphasized that the extent of inner retinal disorganization and outer retinal involvement is the strongest structural determinant of visual outcome in CHRRPE, a correlation that is strikingly exemplified by the present case.

The fluorescein findings in our case are concordant with those described by Shields et al. [4] and by Font et al. [10], who highlighted the dilated, tortuous vascular loops at the disc as a distinctive fluorescein feature of epipapillary CHRRPE. Additionally, in our case the temporally located posterior pole sector illustrated a multimodal signature highly suggestive of chronic inner retinal ischemia: on FA, this region showed subtly reduced capillary detail and dull late staining without frank leakage; on SD‑OCT, it corresponded to abnormally hyperreflective, disorganized inner retinal layers adjacent to broad schitic cavities; and on BAF, it appeared as a homogeneous plaque‑like area of increased autofluorescence. Taken together, these converging findings support the interpretation of long‑standing traction‑related ischemic injury and gliotic remodeling in the retinal sector most exposed to tangential forces exerted by the epipapillary hamartoma.

Regarding multicolor imaging, Kaprinis et al. [17] provided the most detailed characterization to date of CHRRPE, describing the green reflectance (GR) channel as particularly informative in delineating the extent of epiretinal gliosis, inner retinal dragging, and superficial retinal striations, while the near-infrared reflectance channel defined lesion boundaries through peripheral hyper-reflectance at the lesion edges and central hypo-reflectance corresponding to the tumor core. Their three juxtapapillary cases showed "red-shifting" on composite multicolor images where partial pigmentation was present. In our case, the dominant composite multicolor feature is instead a pronounced green-dominant discoloration of the entire mass, a finding reflecting the high relative contribution of the 518 nm GR channel, consistent with the altered spectral reflectance of the fibrous and glial tissue at the vitreoretinal interface that replaces the normal inner retinal structure throughout the lesion. The exceptional size of the hamartoma in our patient renders the GR-dominant signal particularly conspicuous and diffuse compared to the smaller lesions reported by Kaprinis et al. Taken together, BAF and multicolor imaging provide complementary information to FA and OCT in CHRRPE, and their systematic inclusion in the diagnostic workup should be encouraged as part of a standardized multimodal protocol.

An additional noteworthy feature of this case is the presence of a temporally located posterior pole sector showing homogeneous hyperautofluorescence, attenuated capillary detail on FA, and marked inner retinal thinning on OCT. This pattern is compatible with chronic, traction‑related inner retinal ischemia and remodeling. Although ischemic changes have been rarely emphasized in prior CHRRPE series, the extensive and long‑standing vitreoretinal traction in epipapillary lesions may plausibly compromise inner retinal perfusion in the most traction‑stressed sectors, leading over time to tissue loss and increased autofluorescence from unmasked or reorganized RPE [16]. Recognizing this mechanism is important, as it further explains the profound visual loss observed in advanced cases without invoking active neovascular or exudative complications [15,16].

There is no established treatment for CHRRPE, and management is guided by the degree of vitreoretinal traction and macular involvement. Observation is recommended in the absence of progressive visual loss or significant macular traction [2,4]. The role of vitreoretinal surgery remains debated. Early series, including McDonald et al. [8], reported no visual benefit from membrane peeling in adult macular CHRRPE, attributing failure to the inseparability of the glial epiretinal component from the underlying dysplastic retina. More recent surgical series have yielded more encouraging results: Cohn et al. [11] documented visual improvement in the majority of 11 pediatric eyes after pars plana vitrectomy with membrane peeling, and Sun et al. [18], in the largest series to date (15 patients, mean follow-up 5.7 years), reported visual acuity improvement in 93% of cases with anatomical improvement in all eyes.

Across all series, earlier intervention, before irreversible outer retinal disruption, and careful preoperative OCT-based patient selection, are identified as the strongest determinants of a favorable outcome [11,19]. In our patient, BCVA of hand motion, massive epipapillary elevation, full-thickness retinal disorganization, and extensive traction retinal detachment components on OCT suggest long-standing, likely irreversible structural damage, placing this case at the severe end of the surgical risk-benefit spectrum. Accurate surgical planning is currently ongoing. 

These observations collectively suggest that systematic multimodal imaging should be considered standard of care in the diagnostic workup of any suspected epipapillary lesion, as it enables confident diagnosis, exclusion of mimickers, and precise preoperative stratification of structural damage.

## Conclusions

This case highlights that epipapillary CHRRPE may remain clinically silent for years in adulthood and it may present with dramatic visual loss as the first reported symptom, possibly facilitated by unrecognized unilateral progression in a presumably non-dominant eye. Cross-modal correlation between SD-OCT, FA, and autofluorescence is essential not only for diagnosis but also for identifying advanced traction-related complications such as retinoschisis, inner retinal tissue loss, and ischemic remodeling, which carry direct prognostic and surgical implications.
